# Pediatric Critical Care in Resource-Limited Settings—Overview and Lessons Learned

**DOI:** 10.3389/fped.2018.00049

**Published:** 2018-03-16

**Authors:** Tina M. Slusher, Andrew W. Kiragu, Louise T. Day, Ashley R. Bjorklund, Arianna Shirk, Colleen Johannsen, Scott A. Hagen

**Affiliations:** ^1^Department of Pediatrics, University of Minnesota and Hennepin County Medical Center, Minneapolis, MN, United States; ^2^London School of Hygiene & Tropical Medicine, London, United Kingdom; ^3^Walter Reed National Military Medical Center, Bethesda, MD, United States; ^4^Department of Pediatrics AIC Kijabe Hospital, Kijabe, Kenya; ^5^Cardiac Intensive Care Unit of Regions Medical Center, St. Paul, MN, United States; ^6^Department of Pediatrics, University of Wisconsin, Madison, WI, United States

**Keywords:** pediatric critical care, low resource settings, low middle-income country, pediatric intensive care, partnership practice

## Abstract

Pediatric critical care is an important component of reducing morbidity and mortality globally. Currently, pediatric critical care in low middle-income countries (LMICs) remains in its infancy in most hospitals. The majority of hospitals lack designated intensive care units, healthcare staff trained to care for critically ill children, adequate numbers of staff, and rapid access to necessary medications, supplies and equipment. In addition, most LMICs lack pediatric critical care training programs for healthcare providers or certification procedures to accredit healthcare providers working in their pediatric intensive care units (PICU) and high dependency areas. PICU can improve the quality of pediatric care in general and, if properly organized, can effectively treat the severe complications of high burden diseases, such as diarrhea, severe malaria, and respiratory distress using low-cost interventions. Setting up a PICU in a LMIC setting requires planning, specific resources, and most importantly investment in the nursing and permanent medical staff. A thoughtful approach to developing pediatric critical care services in LMICs starts with fundamental building blocks: training healthcare professionals in skills and knowledge, selecting resource appropriate effective equipment, and having supportive leadership to provide an enabling environment for appropriate care. If these fundamentals can be built on in a sustainable manner, an appropriate critical care service will be established with the potential to significantly decrease pediatric morbidity and mortality in the context of public health goals as we reach toward the sustainable development goals.

Critical care is healthcare for the sickest patients. The concept of critical care developed during the poliomyelitis epidemic in the 1920–1950s when the number of critically ill patients led to the development of dedicated areas, “intensive care units,” that provided continuous care and monitoring. Critical care services, with focused attention and expertise provided by medical and nursing staff for patients with multi-organ failure and life-threatening illness, quickly spread worldwide (1). Critical care services were extended to pediatric patients in high income countries (HICs) by the 1950s and early pioneers of critical care in low middle-income countries (LMICs) were only 15 years behind their HIC counterparts, opening the first African intensive care unit in 1969 (2). Nearly 50 years later, however, a huge gap has emerged between the high quality pediatric critical care services available in most HIC settings and the inadequate pediatric critical care services available in most LMIC settings. Nearly 90% of the children who die globally each year below the age of 5 are living in 42 of the poorest countries. In these countries many illnesses and injuries are preventable and treatable diseases are widespread. In a call to action, Niranjan Kissoon described a journey of an African mother seeking care for her ill child filled with fear and frustration (3). The journey elegantly demonstrated how “accident of latitude” often determines whether one lives or dies (4).

## History of Pediatric Critical Care in HICs

The first pediatric intensive care units (PICU) were developed in 1955 in Sweden. This unit treated children with pneumonia and sepsis by extending knowledge gained from the adult ICUs (5). In HICs, PICUs developed rapidly over the subsequent decades with innovations in care and equipment as well as specialization of nursing and medical staff. In the 1990s, the American Academy of Pediatrics and the Pediatric Section of the Society for Critical Care Medicine published guidelines for establishment of pediatric critical care units (6) and for PICU admission criteria (7). The admission criteria were intentionally vague and broad, recommending admission for severe or life-threatening illness and/or illnesses requiring frequent monitoring or intensive intervention (8). The guidelines for establishing a PICU focused on staff training, nursing-to-patient ratio, equipment, and ancillary/support services needed to establish quality critical care. As the specialty advanced, critical care in HICs progressed from treating mostly curable acute, life-threatening illness to provide technologically advanced interventions and complicated coordination of care for patients with complex medical conditions. The interventions provided also evolved from relatively simple and inexpensive therapies, such as fluid resuscitation, oxygen, and medications to complex and expensive therapies, including organ transplantation, dialysis, and extracorporeal membrane oxygenation. The evolution of pediatric intensive care in HICs is a model for the development of the same care in LMICs; starting with inexpensive, simple, lifesaving care, and increasing capabilities as resources and training become available.

## History of Pediatric Critical Care in LMICs

The details of the first PICU and the development of specialized pediatric critical care services in LMICs are not as well documented as in HICs. A systematic review aimed at estimating ICU capacity in low resource settings reported a lack of critical care beds and pediatric intensive care services (9). Millennium Developmental Goal Four (MDG4) targeted a two-thirds reduction in childhood mortality in children less than 5 years of age by the year 2015. To achieve MDG4, UNICEF and WHO focused on the outpatient/ambulatory setting, aiming to improve hygiene, nutritional support, breastfeeding, and immunizations (10). The Integrated Management of Childhood Illness program was established to guide outpatient care. Based on these guidelines, an estimated 20% of children seen in the ambulatory setting met criteria for referral to a hospital for escalation of care (10). Hospital case mortality rates have decreased only slowly from the 1990s to early 2000s, highlighting the need for improved care of the sickest hospitalized children, including triage, emergency, and critical care services (8).

In response to the need for improved triage and emergency care, the WHO developed the Emergency Triage, Assessment, and Treatment (ETAT) guidelines (11). These guidelines emphasize the tenets of pediatric critical care with early recognition of children who needed immediate care and hospitalization. Implementation of these guidelines in a Malawi hospital decreased the mortality rate by half (10–18 to 6–8%) from 2001–2006 (12).

The WHO also published and updated *The Pocketbook of Hospital Care for Children for the Management of Common Illnesses with Limited Resources* (13), which provides many appropriate clinical guidelines for nurses and physicians caring for hospitalized children in these settings. The development of these guidelines and improvements in staff training clearly demonstrated that advances can be made in caring for critically ill children in LMICs by appropriate triage and rapid treatment using relatively inexpensive modalities, such as fluids, oxygen, and antibiotics.

## Current State of Pediatric Critical Care Services in LMICs Settings

In LMIC settings, the burden of pediatric mortality remains high and a largely undocumented burden of critical illness exists (14). The “three delays” model, first developed to explain why maternal deaths occur may be applied to identify factors that are significant contributors to childhood mortality in LMICs (15). The three delays are (1) delay in deciding to seek care, (2) delay in reaching the appropriate facility, and (3) delay in receiving quality care at the facility. Examples of the third delay, with regards to pediatric critical care services in LMIC settings, are a lack of designated intensive care units with adequate numbers of healthcare staff trained to care for critically ill children, and rapid access to necessary medications, supplies, and equipments. Effective critical care in HICs relies on having a dedicated area, where close observation and frequent monitoring of pediatric patients leads to the provision of rapid, timely, appropriate interventions.

Despite the published guidelines for triage and fundamentals of care described above, the recognition and ability to provide rapid interventions still remains largely absent in many LMIC settings. Infections, such as sepsis, pneumonia, and malaria continue to have a high mortality and 90% of childhood trauma deaths occur in LMICs (16).

There are also significant disparities in pediatric critical care capabilities within regions of any given LMIC (8). In some African countries, university and private hospitals are capable of providing pediatric critical care comparable to PICUs in HICs (8). In large cities throughout China, India, South Africa, South America, and the Middle East, emergency and intensive care services are often similar to HIC settings (8). The majority of hospitals in LMICs, however, do not have a designated PICU with pediatric trained nursing staff, adequate nurse to patient ratios to care for critical patients, appropriate equipment, monitoring capability, or ancillary support (9). Most pediatric critical care is performed in mixed adult-PICU and the majority of these units would be considered the lowest level ICU (pediatric level 2) (17). PICUs that are established in LMIC’s are typically staffed by general pediatricians and lack specialized services.

Without a formal pediatric critical care training, curriculum, or certification process, there is a wide variation in understanding skills and care provided. Finally, rural hospitals and clinics in these same countries are not equipped with even the basic resources, such as oxygen, resuscitation equipment, and medication to provide pediatric emergency care, and appropriately trained staffs are rarely available (15).

## The Arguments Against and the Case for PICUs in LMICs

Given the perceived high cost of developing and maintaining critical care services, questions naturally arise regarding the advisability or feasibility of establishing critical care units in LMICs. Indeed, financial instability was reported in 35% of established PICUs in the LMICs vs 2.6% of the HICs (13). One could argue that resources would be better employed in addressing the most common primary public health problems facing these communities. In addition, since many of the essential aspects of basic emergency care are not present in LMICs, provision of quality intensive care can be difficult (15). Critical care services in HICs involve a well-coordinated system including three components: triage, emergency medical care and intensive care. Such a comprehensive system is largely unattainable in many LMICs. Yet, despite the strain on resources and the difficulty in establishment of complex systems of care, there is a need to manage children with sudden, serious reversible disease in all settings (14).

An argument for developing critical care services is the potential for these services to translate to improvements in hospital care for all patients (18). Many of the conditions that contribute to the burden of disease in LMICs, such as dehydration and respiratory distress, can be mitigated through prompt, simple treatments (14). Pediatric emergency and critical care services do not need to be expensive, nor excessively dependent on complex technology (19). Critical care services can be utilized to improve outcomes if combined with a focus on community recognition of serious illness, early access to care, referral, and safe transport (15). A robust triage system, the first component of critical care, is still formally lacking in many hospitals in LMICs despite the WHO ETAT recommendations (19). The South African Triage Scale (SATS), for example, employs clinical signs and a triage early warning score to assist in the early identification of acute illness. Use of the SATS has led to better use of hospital resources and earlier discharge (8). Implementing simple tools and interventions such as these, can make a significant difference in the outcomes of critically ill children, supporting their use in LMICs.

Finally, a key argument for the provision of critical care services in resource-limited settings lies within an ideology that all human beings belong to a single community, based on a shared morality, and is based on three principles: the worth of individuals, equality, and the existence of obligations binding to all (8).

## Basics of Developing a PICU in a LMIC: Lessons Learned

Preparing to set up a pediatric critical care unit in a LMIC setting requires planning, vision, specific resources, and most importantly investment by the nursing and permanent medical staff. While PICUs in LMIC settings may develop organically, led by national experts within a country, experts from HICs may be asked to provide focused training. It is essential that these visiting experts recognize the importance of collaboration with local medical staff. A needs assessment performed in collaboration with the local team provides important information to guide appropriate establishment of medical services and ensures that programs established are valued by the medical staff and community, making them more likely to be sustainable.

It is essential to recognize that preconceptions can be a barrier to developing pediatric critical care in resource-limited settings. An extreme example is one of “Medical Colonialism,” a term first used by two medical students in 1987 to describe how they were allowed to perform procedures in a LMIC that they would not be allowed to perform in their home country (20). “Medical colonialism,” as a concept, can be more subtle. Resentment and distrust may develop if medical personnel from HICs present themselves as knowing “what is best” for those in LMICs despite being visitors. To avoid this undesirable situation, it is critical to listen to, recognize and respect the expert guidance of the local medical staff, nurses, and healthcare workers and form a true partnership.

Borrowing from the work done by Dr. Paul Farmer and his model of four components required to solve epidemics, we have provided examples of lessons learned in developing pediatric critical care services in resource-limited settings (21, 22). The four S components of Dr. Farmer’s model are: (1) staff: properly trained and compensated healthcare professionals; (2) stuff: appropriate medical equipments; (3) space: a clean environment to treat patients; and (4) systems: the infrastructure and logistical organization to provide the services. Although Dr. Farmer’s model may not be fully applicable to development of sustainable systems of health care, it provides a contextual framework for discussion of the issues encountered and examples of pitfalls to avoid when developing pediatric critical care services.

### Staff: Properly Trained and Compensated Healthcare Professionals

Critical care depends on a team of dedicated, well-trained, and compensated medical and nursing staff. The establishment of a standardized pediatric critical care curriculum and certification for healthcare professionals greatly improves provision of appropriate care. A specialized certification is not currently available in most low resource settings; however, standardized training should continue to be a focus of improving care. Trained nurses are essential to providing appropriate critical care services. Nurse roles in the intensive care unit include patient evaluation, medication administration, data collection and assessment, evaluation of the effectiveness of interventions, and communication with the health care team. There are often obstacles to establishing stable critical care nursing (see Table 1). It cannot be over-emphasized that recommended nurse to patient ratios should be at least 1 nurse to every 3–4 patients in the PICU setting, which may be a dramatic change from the 1:10–30 nurse to patient ratio (PC authors) in many LMIC hospital wards.

**Table 1:** Obstacles to establishing critical care nursing.

Lack of hospital and nursing administration understanding of nursing role in critical care
Lack of funding for specialized training
Inability to provide adequate staffing for ICU nurse to patient ratios
The traditional role of nurses in the hospital including level of education, experience in critical care, cultural norms, and comfort surrounding communication with and respectfully challenging a physician’s orders
Inadequate compensation to encourage retention
Allowing nurses who are fully trained and invested to be allowed to remain in the pediatric intensive care units without frequent rotation and reassignment
The need for well-trained nurses to work as preceptors and mentors to new staff

Focused team-based training is essential in establishing a PICU. Early in the partnership it is important to identify gaps in staff knowledge or skills. Performing a need assessment can provide a template to guide training and meet the objectives of the program. A necessary first step is to establish a solid foundation of basic critical care knowledge and skills. Early training should focus on basic life support skills, recognition of age-specific normal vital signs, input/output fluid balance calculation, and continuous monitoring of the cardiorespiratory system. Recognizing abnormalities in basic physiologic parameters is the first step in a chain of actions to improve outcomes in the PICU. Nurses who typically work in adult ICUs should receive pediatric-specific training focused on how to care for infants and children, knowledge of age-dependent vital signs, and the key differences in resuscitation of pediatrics patients. Simple resources, such as posters or binders with age appropriate vital signs, relevant assessments, and basic therapies are useful while establishing a new PICU. Once basic skills are mastered, it is natural to develop more advanced resuscitation skills. Training can progress to include goal-directed therapy and may include tracheal intubation, mechanical ventilation, use of vasopressors, and placement of central lines. Regular mock codes, critical cases review, and simulation not only reinforce training, but also provide team training and establish the importance of teamwork in the PICU.

A complementary set of skills and knowledge that can have great impact on sustaining a program are those associated with quality improvement (QI), teamwork and leadership. QI provides essential skills to monitor and make adjustments to the program over time as skills and knowledge change, equipment decisions are made, and to continually improve outcomes. As skills in caring for critically ill children are established, a parallel set of skills in teamwork and leadership also become essential in the development of pediatric critical care program. To complement formal teaching, physicians, nurses, and other ICU staffs will benefit from the guidance of mentors to model and help to develop effective teamwork and leadership skills. As critical care services are established in LMICs, mortality rates may remain high while quality care is developed. Therefore, providing emotional support for staff where issues, such as staff turnover, emotional fatigue, and burnout are at epidemic proportions is essential to the well-being of those working in the PICU. Support might include developing the capacity to provide services, such as event debriefing, when mortality or morbidity occurs in the PICU.

### Stuff: Appropriate Medical Equipment

In addition to staffing, medical equipment has an important role in pediatric critical care. When developing critical care services for children in LMICs, a step-wise approach to introducing equipment is an effective way to build a sustainable service without overwhelming the system. Use of low cost, simple technologies and techniques early in the development of critical care can be lifesaving, and improve quality of care in a sustainable long-term manner (19). Resuscitation kits containing an appropriate-sized neonatal and pediatric bag valve mask systems, adrenaline, cannulas, glucose containing fluids, and normal saline should be readily available in the PICU. Respiratory support is an essential aspect of pediatric care. Non-invasive positive pressure ventilation, including bubble CPAP, is likely a safer and more sustainable respiratory support option than ventilators. Non-invasive ventilation is preferred for many reasons, including conservation of oxygen, decreased need for sedation, decreased equipment/maintenance cost, use of battery power sources, and less monitoring needs. The use of “home” ventilators with internal compressors, instead of “hospital” ventilators that rely on high pressure air and oxygen sources to function, can conserve oxygen and resources. Since oxygen may be supplied in tanks, knowing how much oxygen is available is important. Some ventilators will deplete a large oxygen tank in less than an hour.

Using inexpensive, but appropriate, substitutes for costly medical equipment is a way to establish critical care services without the excessive cost found in some HIC. Examples of low cost medical equipment substitutes include adapting plastic water bottles as spacers for inhaler therapy and the use of sterile nasogastric tubes as umbilical vein catheters (19, 23, 24). When purchasing equipment, the availability in the LMIC or region should be considered, so that it may be maintained and repaired locally (19). Additionally, buying equipment locally supports the regional economy while providing a much-needed service to the hospital.

### Space: A Clean Environment to Treat Patients

Providing critical care requires physical space in all settings. Caring for critically ill patients requires more space than caring for non-critically ill patients due to the space needed for equipment and higher number of medical personnel needed per patient.

Overestimating the number of patients that can be cared for in a given space is common. Crowded care areas can create an unorganized work environment that may promote infection, cause difficulty locating necessary equipment, and limit medical personnel and family access to patients. Due to the lack of infrastructure, such as pressurized gases and vacuum, more space may be required in LMICs for equipment that is not needed in HICs. Examples of extra equipment in LMICs may include oxygen tanks, oxygen concentrators, air compressors, and suction machines in addition to monitors, IV pumps, and ventilators that are needed to support the critically ill child in all environments.

### Systems: The Infrastructure and Logistical Organization to Provide the Services

Having support to develop pediatric critical care services from the local leadership, both within the organization and from various levels of government, is essential to the success of the program. Support is necessary to develop and retain trained medical personnel.

Additionally, having a biomedical team that provides a reliable way to replace and repair equipment and the infrastructure to maintain the space and facility is invaluable. When supportive leadership is absent, trained personnel may end up leaving for other opportunities, equipment may fall into disrepair, and the facilities will become unusable. Since no pediatric critical care service works in isolation, relationships between the PICU services and the rest of the organization must also be clarified. Relationships are particularly essential between PICU and emergency medicine, anesthesiology, radiology, and surgery, but good working relationships with all the medical and surgical services that will interact with PICU are important.

Finally, in any medical environment that cares for critically ill children, difficult decisions over the use of limited resources will occur. The most difficult question is often the dilemma of “what can be done, what should be done and what will be done.” Those making the decisions in a resource-limited setting need to be aware of anticipated outcomes for specific illnesses and should avoid the use of limited resources on patients that have a terminal illness (19). Additionally, long-term use of limited resources, such as pumps and ventilators needs to be considered part of resource stewardship. As these difficult situations are inevitable, admission and discharge guidelines should be established early, based on local experience and outcomes, and updated as outcomes change (20). When beds and supplies are limited, PICU admissions should be limited to illness and injury that are reversible or curable, such as shock secondary to dehydration, anemia, or malaria, and survivable trauma. Having admission criteria based on outcomes will prevent the use of limited critical care resources for children with terminal or untreatable conditions or for those unlikely to benefit from treatments (19). Some examples of situations that may deplete medical resources without benefit to the patient, include complex congenital heart disease in the absence of a cardiac surgery program, malignancies without effective therapy, and patients in persistent coma requiring mechanical ventilation. It is also important to ensure families don’t utilize all of their resources, financial, or otherwise, since the health status of many LMIC individuals and families is closely tied to their ability to stay out of poverty (19). Therefore, ensuring financial support is available to a family should be part of the treatment plan for critically ill children in LMICs. Finally, local medical personnel are invaluable in understanding the limits of their system, knowing when to stop or limit resuscitation, or limit the use of supplies and medicines that might be considered unhelpful or wasteful. Listening to local medical providers is an important aspect of respecting their expertise and culture.

## Conclusion

The principal goal of developing critical care services in LMICs is to progressively improve the outcomes of children presenting with a serious or life-threatening illness and to do it in a sustainable manner. This requires working together as partners with LMIC healthcare providers in a respectful and nurturing environment. A thoughtful approach to developing pediatric critical care services starts with fundamental skills and knowledge, inexpensive, but effective equipment, and supportive leadership. These fundamentals can be built on in a sustainable manner, having the potential to significantly decrease pediatric morbidity and mortality in LMICs through excellent, but appropriate critical care in the context of public health goals as we reach toward the 2030 SDGs.

## Author Contributions

TS substantial contributions to the conception or design of the work, or the acquisition of background articles and topics to be covered. Drafted work and revised it critically for important intellectual content. Provided approval for publication. Agreed to be accountable for all aspects of the work in ensuring that questions related to accuracy or integrity of any part of work are appropriately investigated and resolved. AK and CJ drafted work and added content critically for important intellectual content. Provided approval for publication of content. LD revised work and added content critically for important intellectual content. Provided approval for publication of content. AS drafted and revised the work and added content critically for important intellectual content. Provided approval for publication of the content. SH drafted work and revised it critically for important intellectual content. Provided approval for publication. Agreed to be accountable for all aspects of the work in ensuring that questions related to accuracy or integrity of any part of work are appropriately investigated and resolved. AB drafted work for the article and critically edited, reviewed and approved the final version.

## Conflict of Interest Statement

The authors declare that the research was conducted in the absence of any commercial or financial relationships that could be construed as a potential conflict of interest.

## References

[1] Marshall JC Bosco L Adhikari NK Connolly B Diaz JV Dorman T What is an intensive care unit? A report of the task force of the World Federation of Societies of Intensive and Critical Care Medicine. J Crit Care (2017) 37:270–6. 10.1016/j.jcrc.2016.07.015 27612678

[2] Meiring Pde V Lumsden JD Morrison AG Furnham LA An intensive care unit in a provincial general hospital. S Afr Med J (1969) 43(26):806–10. 5801576

[3] Kissoon N Out of Africa – a mother’s journey. Pediatr Crit Care Med (2011) 12(1):73–9. 10.1097/PCC.0b013e3181ce74ef 20068505

[4] Fowler RA Adhikari NK Bhagwanjee S Clinical review: critical care in the global context – disparities in burden of illness, access, and economics. Crit Care (2008) 12(5):225 10.1186/cc6984 19014409 PMC2592728

[5] Epstein D Brill J. A history of pediatric critical care medicine. Pediatr Res (2005) 58(5):987–96. 10.1203/01.PDR.0000182822.16263.3D 16183804

[6] Guidelines and Levels of Care for Pediatric Intensive Care Units. Committee on Hospital Care of the American Academy of Pediatrics and Pediatric Section of the Society of Critical Care Medicine. Pediatrics (1993) 92(1):166–75. 8516070

[7] Guidelines for Developing Admission and Discharge Policies for the Pediatric Intensive Care Unit. Pediatric Section Task Force on Admission and Discharge Criteria, Society of Critical Care Medicine in conjunction with the American College of Critical Care Medicine and the Committee on Hospital Care of the American Academy of Pediatrics. Crit Care Med (1999) 27(4):843–5. 10321680

[8] Turner EL Nielsen KR Jamal SM von Saint Andre-von Arnim A Musa NL. A review of pediatric critical care in resource-limited settings: a look at past, present, and future directions. Front Pediatr (2016) 4:5. 10.1080/09540121.2016.1139043 26925393 PMC4757646

[9] Murthy S Leligdowicz A Adhikari NK. Intensive care unit capacity in low-income countries: a systematic review. PLoS One (2015) 10(1):e0116949. 10.1371/journal.pone.0116949 25617837 PMC4305307

[10] Baker T. Pediatric emergency and critical care in low-income countries. Paediatr Anaesth (2009) 19(1):23–7. 10.1111/j.1460-9592.2008.02868.x 19076498

[11] World Health Organization. Emergency Triage Assessment and Treatment (ETAT) Manual for Participants. Geneva, Switzerland: World Health Organization (2005).

[12] Molyneux E Ahmed S Robertson A. Improved triage and emergency care for children reduces inpatient mortality in a resource-constrained setting. Bull World Health Organ (2006) 84(4):314–9. 10.2471/BLT.04019505 16628305 PMC2627321

[13] World Health Organization. Pocketbook of Hospital Care for Children Guidelines for the Management of Common Illnesses with Limited Resources. Geneva: World Health Organization (2013).

[14] Tripathi S Kaur H Kashyap R Dong Y Gajic O Murthy S. A survey on the resources and practices in pediatric critical care of resource-rich and resource-limited countries. J Intensive Care (2015) 3:40. 10.1186/s40560-015-0106-3 26457187 PMC4599333

[15] Thaddeus S Maine D. Too far to walk: maternal mortality in context. Soc Sci Med (1994) 38(8):1091–110. 10.1016/0277-9536(94)90226-7 8042057

[16] Baker T. Critical care in low-income countries. Trop Med Int Health (2009) 14(2):143–8. 10.1111/j.1365-3156.2008.02202.x 19207174

[17] Rosenberg DI Moss MM American College of Critical Care Medicine of the Society of Critical Care M. Guidelines and levels of care for pediatric intensive care units. Crit Care Med (2004) 32(10):2117–27. 10.1097/01.CCM.0000142704.36378.E9 15483423

[18] Amoateng-Adjepong Y Caring for the critically ill in developing countries – our collective challenge. Crit Care Med (2006) 34(4):1288–9. 10.1097/01.CCM.0000208352.74208.75 16550100

[19] Slusher T Bjorklund A Aanyu HT Kiragu A Christo P The assessment, evaluation and management of the critically ill child in resource limited international settings. J Pediatr Intensive Care (2016) 06(01):066–076. 10.1055/s-0036-1584677 PMC6260265 31073427

[20] Holt TA Adams TJ Medical colonialism. J Med Ethics (1987) 13(2):102 10.1136/jme.13.2.102

[21] Farmer P The Ebola Suspect’s Dilemma. Keynote Address for the MacLean Prize Lecture. Chicago, IL (2017).

[22] Farmer P Taking up the Challenges of Poverty: Why Accompaniment Matters. Notre Dame: Lecture delivered at Kellogg Institute for International Studies (2016)

[23] Simulation Use for Global Away Rotations (SUGAR). (2016). Available from: http://sugarprep.org/pearls/ (accessed January 27, 2018).

[24] Bensman RS Slusher TM Butteris SM Pitt MB On Behalf of the Sugar Pearls Investigators Becker A Creating online training for procedures in global health with PEARLS (Procedural Education for Adaptation to Resource-Limited Settings). Am J Trop Med Hyg (2017) 97(5):1285–8. 10.4269/ajtmh.16-0936 28820680 PMC5817738

